# The influence of maturation, fitness, and hormonal indices on minutes played in elite youth soccer players: a cross-sectional study

**DOI:** 10.1186/s13102-022-00480-8

**Published:** 2022-05-17

**Authors:** Ebrahim Eskandarifard, Hadi Nobari, Filipe Manuel Clemente, Rui Silva, Cain C. T. Clark, Hugo Sarmento, António José Figueiredo

**Affiliations:** 1 Department of Exercise Physiology, Faculty of Sport Sciences, University of Isfahan, Isfahan, 81746-7344 Iran; 2 Department of Motor Performance, Faculty of Physical Education and Mountain Sports, Transilvania University of Braşov, 500068 Braşov, Romania; 3 Department of Exercise Physiology, Faculty of Educational Sciences and Psychology, University of Mohaghegh Ardabili, Ardabil, 56199-11367 Iran; 4 Sports Scientist, Sepahan Football Club, Isfahan, 81887-78473 Iran; 5 Escola Superior Desporto e Lazer, Instituto Politécnico de Viana do Castelo, Rua Escola Industrial e Comercial de Nun’Álvares, 4900-347 Viana do Castelo, Portugal; 6 Delegação da Covilhã, Instituto de Telecomunicações, 1049-001 Lisbon, Portugal; 7 Centre for Intelligent Healthcare, Coventry University, Coventry, CV1 5FB UK; 8 Research Unit for Sport and Physical Activity, Faculty of Sport Sciences and Physical Education, University of Coimbra, Coimbra, Portugal

**Keywords:** Football, Young, Maturation, Performance, Playing time, Talent development, V̇O_2max_

## Abstract

**Background:**

The main purpose of this study was to investigate the relationships between minutes played (MP) with maturity status, fitness, and hormonal levels, and to quantify their influence on minutes played.

**Methods:**

Twenty-four elite youth soccer players under-16 years participated in this study, over a full-season period. Anthropometric measures, maturity status, hormonal and physical fitness levels were collected. Participants were monitored during the season. After the end-season, players were assessed in 6 different tests over a four-day period.

**Results:**

The maximum oxygen consumption (V̇O_2max_) was strongly correlated with MP (r = 0.75), maturity offset (r = 0.52), and countermovement jump (r = 0.53). Multiple linear regression explained 76% of MP (F (8, 15) = 6.05, p = 0.001), with an R2 of 0.76. Moreover, Growth hormone (GH) and V̇O_2max_. were the most influential factors in MP (F (2, 21) = 17.92, p ≤ 0.001), with an R2 of 0.63.

**Conclusion:**

High levels of GH and V̇O_2max_ have a preponderant role in MP by elite youth soccer players, it appears to be more pragmatic to consider other contextual dimensions, as they can impact selection for competition and minutes of participation in a match.

## Background

Most soccer academies worldwide, team categorization and stratification are based on date of birth (chronological age) [1], where the maturity status (biological age) of young soccer players is often overlooked [2]. The differences in maturity between players of the same chronological age has been extensively researched [3], as well as the preferences for early maturing players in relation to their later maturing counterparts [4, 5]. Children are expected to deal with a growth spurt, coincidental with the onset of puberty, which happens in different momentums and at different rates [6], the highest record of this rapid growth phenomenon is also known as the peak height velocity (PHV) (usually at ~ 14 years old, for males) [7]. There are several methods of controlling the maturation status of young players, such as the assessment of skeletal age through the Fels or TW3 method [8], however, requires expensive equipment and expose the children to radiation [9]. Fortunately, there are other ways in which the prediction of the maturity offset (age at PHV) can be achieved, by simply measuring the weight, standing height, sitting height, and leg length, and applying a simple calculation [10]. However, it must be highlighted the fact that errors of one year may occur 95% of the times that the calculation of maturity offset is made [11].

In using such methods, coaches are better able to perform talent identification, selection and development [2, 12]. Indeed, the maturity variability that may exist within the same team must be acutely considered, as the players in the same age-category team normally present with great differences in maturity levels [13, 14].

At the onset of puberty, it is expected that the hormonal system begins to operate without major sex differences in the rate of growth and maturation or in the expression of physical qualities [15]. However, as they are close to reaching the growth spurt, or at PHV, a clear differentiation between sex in the expression of physical qualities is demonstrable [16, 17]. The activity of circulating hormones, such as the growth hormone (GH) and insulin-like growth factor 1 (IGF1), are expected during this phase of growth, often resulting in augmented levels of fitness [18–20]. Furthermore, it has been reported that soccer practice in children, at the onset and until the end of puberty, can yield increased levels of circulating GH and IGF1 concentrations, which are associated with greater jump performance [21]. Moreover, because of the anabolic activity during growth, it is expected that early maturers have greater neural and structural stimuli, resulting in the maximization of existing qualities, thus surpassing the later maturers [7, 22, 23]. In fact, it has been shown that early maturing players outperformed later maturers in aerobic capacity, speed, strength, and power [14, 24, 25]. However, late maturing soccer players appear to benefit more than early maturers in terms of balance and body control, as maturational advanced players have to deal with an augmented coordination awkwardness [26].

Talent identification and selection in soccer is generally based on traditional methods, where it is mistakenly believed that early maturing players are the ideal short- and long-term prospects. The inconvenient truth is that early maturing players are preferred over late maturing players, mainly due to their taller and stronger characteristics, thus excluding their smaller colleagues [6, 12, 27]. Furthermore, there is no consensus in terms of the influence of different maturation statuses on match performance [23, 28]. On one hand, it has been demonstrated that early maturing soccer players cover greater total distances and high intensity running distances than late maturing counterparts [23]. On the other hand, it has been reported that early maturers present the greatest high intensity running distances in relation to their late maturing peers [28]. Despite equivocality in the literature, late maturing players seem to have a greater chance of success in attaining elite level status [3, 29].

Another pertinent issue regarding the differences related to opportunities given to early and later maturers is the time of play and selection for competition. There exists an observable trend towards selection of the stronger and taller players (early maturers) as players are closer, or at, PHV [13], in an attempt to ensure better match results, as opposed to the developmental process [30]. These practices knowingly lead to a greater risk of dropout by late maturing players [29, 31]. However, there is still a lack of evidence supporting the influence of maturity status on play time. Indeed, Goto et al. [23] revealed that more advanced maturing players had more opportunities to play in a match in U9 and U10 teams, although, the same trend was not observed at U11 and U14 teams.

Time of play and selection for competition may be influenced by the level of maturation, the activity of circulating GH, as well as the level of fitness of young players that are at the critical stages of adolescence. Whilst the influence of maturation levels on time of play has begun to be investigated [23], the evidence is too small and inconclusive. Interaction between maturation status, operating GH, and level of fitness, may yield more precise information for talent identification and selection.

Therefore, the aims of this study were twofold: (i) to analyze the relationship between minutes played (MP) in soccer and the skeletal age, maturity offset, maximal oxygen uptake (V̇O_2max_), fatigue index in repeated sprint test, countermovement jump (CMJ) performance, GH, and IGF1 levels, respectively; and (ii) to investigate to what extent the aforementioned variables can explain the variance in MP. It is hypothesized that at U16 soccer teams, advanced maturity and physical fitness status will influence player’s playing times at a great extent.

## Methodology

### Participants

Twenty-four elite youth soccer players (Mean ± Standard deviation (SD); age: 15.61 ± 0.27 years; height: 176.50 ± 5.23 cm; body mass: 60.97 ± 8.29 kg; skeletal age: 16.65 ± 0.84 years; maturity offset: 1.97 ± 0.43 years; V̇O_2max_, 49.84 ± 3.48 ml kg^−1^ min^−1^) who played in the Iranian U16 Premier League participated in this study. Considering the field position, the sample was composed by 2 goalkeepers, 9 defenders, 5 central midfielders, 4 wingers, and 4 attackers. The inclusion criteria were: (1) at least 4 years of experience playing soccer; (2) active and regular participation in all stages of the study; (3) they did not use any supplement that could affect growth and maturation; and, (4) participants were not permitted to perform additional exercise/training regimens. The exclusion criteria were: (1) not participating in 80% of competitions (formal and no formal) and exercises of the whole season; (2) did not attend one of the medical or physical tests of the study. This study was approved by the Ethics Committee of University of Isfahan, as well as operating in accordance with Declaration of Helsinki. Before participating in the study, players and their parents/guardians were notified about risks and benefits of this study, and signed informed consent and assent, respectively.

### Sample size

Based on a previous study that highlighted large to very large correlations between physiological variables in young soccer players [32, 33], we computed the sample size necessary to achieve a power of, at least, 0.90. Accordingly, using a correlation with the point biserial model hypothesis, expected largely to very large correlations, and an α error probability of 0.05 and 1-β err probability, 21 participants would be required to achieve the aforementioned power. The sample size was calculated using G-Power software (University of Düsseldorf, Düsseldorf, Germany).

### Experimental approach to the problem

This research was conducted as a quasi-experimental and cohort study which was performed on a cross-sectional basis, yielding practical results. This study had two purposes: (i) to elucidate the relationship between playing time in soccer and the skeletal age, maturity offset, V̇O_2max_, fatigue index in repeated sprint test, CMJ performance, GH, and IGF1 levels, respectively; and (ii) to investigate to what extent the aforementioned variables can explain the variance in playing time. Players took part in a formal or non-formal game every week throughout the season. Participants were monitored during the season, and tests were performed upon completion of the competitive season. The timeline of the study can be found in the Table 1.

**Table 1:** During control in season and assessment

Year	2018					2019				Total
Months	August	September	October	November	December	January	February	March	End of March	
Phases	First Preparation phase (8 week)		Regional Games			Best of south Region	Second Preparation phase (6 week)	Best of Iran (National)	Assessment	
Official Games			4	4	4	5		4	4 days	21
Non Official Games	2	4					6			12

The players were assessed over four days, which commenced following three days of rest from the last training session. On the first day, players underwent anthropometric measurements, blood tests, and EOS imaging. On the second day, the CMJ test was used to assess the lower-limb power. On the third day, anaerobic power was tested using seven repeated sprint tests (7RST), and on the fourth day, the Yo-Yo Intermittent Recovery Test level 1 was performed to estimate the aerobic capacity of the participants. All tests were performed in the same weather conditions (21–23 °C temperature and 50% humidity) [34] in the morning period to reduce the impact of diurnal variation.

### Anthropometric measurements

To measure the standing height, participants were unshod, with the heels, hips, shoulder blades, and back of the head as close as possible to the stadiometer, with feet placed beside each other. For sitting height, participants sat on a 50 cm bench and were instructed to bring their buttocks as close as possible to the stadiometer, holding their upper body straight and placing their hands on their feet, at which point, their height assessed. The distance between the highest point of the head and the bench was calculated as sitting height. For this measurement, SECA Model 213, Germany, was used with an accuracy of 5 mm. For measuring maturity offset and age at PHV, we used the following formula: Maturity offset =  − 9.236 + 0.0002708 (leg length × sitting height)—0.001663 (age × leg length) + 0.007216 (age × sitting height) + 0.02292 (Weight by Height ratio), R = 0.94, R2 = 0.891, and SEE = 0.592) and for leg length = Standing Height (cm)—Sitting height (cm) [35]. Participants wore only minimal clothing for the measurement of weight, which was conducted using electronic weighing scales, (SECA, model 813, England), with an accuracy of ± 0.1 kg. All anthropometrics measurements were performed in the morning [36, 37]. For standing and sitting height, the inter and intra-observer technical error of measurement was less than 5%, and for weight, it was less than 3%.

### Skeletal age

To assess the skeletal age, posterior-anterior radiographs of the left hand-wrist were taken using the EOS imaging system to obtain a 2D X-ray. The EOS imaging system delivers a radiation dose which is 50 to 85 percent lower [34, 36, 38] than a digital radiography system X-ray, whilst also providing enhanced image quality [39–41]. The Fels method was used to determine the skeletal age according to a series of criteria which yields a higher reliability than other methods in skeletal assessment [8]. The Fels method has special criteria for each bone of the hand-wrist, as well it utilizing ratios of width epiphyses and width diaphysis of radius, Ulna, Metacarpals, and phalanxes, after determining the ratio and maturity grade of each criterion, those numbers are entered into Felshw 1.0 software to calculate skeletal age and standard error. To calculate the chronological age used in the Fels method, the difference between birth date and the date of wrist radiography was calculated up to two decimal places. When the skeletal age of the players was assessed, we used the following equation for classification of the maturity status of participants: Maturity Status = Skeletal Age—Chronological Age, and ultimately, if the maturity Status was more than + 1 participant will be considering early mature, if the maturity status was less than − 1, the participant will be defined as late mature and, if the maturity status wasis between ± 1, the participant will be defined an average mature. With skeletal age evaluations, the intra-observer error was less than 0.21 year.

### Blood analysis

In order to determine the levels of GH and IGF1, after at least 12 h of fasting and 72 h after the last training session, 10 ml of blood was drawn from the antecubital fossa of each participant, at 8 am, in the Al-Zahra Hospital laboratory, where the samples were immediately centrifuged in the laboratory [42, 43]. Subsequently, serum was removed from the blood and used to measure the relevant hormones. To measure GH and IGF1, the Chemiluminescence method (ICMA) and IMMULITE system (2000xpi Systems, company SIMENS Germany). For GH (REF: L2KGRH2; lot 171), the analytical sensitivity of the kit was 0.01 ng/mL, with an average inter-assay coefficient of variability (CVs) of 3.76%, for a mean concentration of 7.3 ng /ml. For IGF1 IGF1 kits; REF: L2KGF2 and lot: 571), the sensitivity was 13.3 ng/ml, with an average inter-assay CVs of 4.4%, for a mean concentration of 379.83 ng /ml.

### Countermovement Jump test

To assess explosive lower-body power [44], we used the CMJ test. All participants undertook a standardized warm-up, consisting of 10 to 15 min. Movements included jogging, followed by 5 to 6 sprint-specific drills, CMJs, horizontal bounds, and vertical hops. Before starting to test each player performed one or two trial jumps for familiarization. For testing, the participant stood on the contact mat, with hands on the hips and ~ 90-degree bend in the knee, and then jumps vertically with maximum power on the command of the examiner. After 5 min of recovery, the participant repeats this test and the best performance, in centimeters, was recorded [45, 46]. The intra-class coefficient (ICC) was evaluated for reliability and found to be 0.84.

### Repeated sprint test

The Bangsbo repeated sprint test, also known as the 7RST test, was used for measuring anaerobic power. According to the test protocol, each participant must perform 7 curved speeds, with a 25-s active recovery time between each sprint. Participants warmed up for 15 min, including jogging, dynamic stretch, ABC training, and sub maximal speed, under the supervision of a fitness instructor. For the test, the participant starts running at their maximum speed on the signal of the examiner, to the end of the demarcated path, followed by 25 s of active recovery. After 25 s, the participant runs again at maximum speed, and this procedure is continued until the 7th run. The difference between the slowest record and the fastest record was calculated as fatigue index [47]. In instances where a participant loses one of these 7 performances for a reason such as slipping, an average of 6 other records was used. This test was performed by photo-finish sensors, which were located at the beginning and end of the path, and a stopwatch was used to measure the 25-s recovery time. This Bangsbo repeated sprint test and CMJ test, respectively, were measured using the Newtest Powertimer 300-series testing system (Newtest, by, Finland). The ICC was evaluated for reliability and found to be 0.79.

### Yo-yo Intermittent Recovery Test

The Yo-Yo Intermittent Recovery Test level 1 (IR1) was used to test the aerobic capacity of players. In this test, each athlete ran a 20 m’ distance back and forth between points, after standardized warm-up (as reported above), according to standardized protocol [48]. Briefly, the first stage is conducted at 10 km/h, and the speed of every level increases by 0.5 km/h. This test continues until the participant fails twice to reach the necessary points. The level at which the participant failed the test and distance covered was recorded, and V̇O_2max_ was calculated based on the following formula: V̇O_2max_ (ml.kg^−1^.min^−1^) = IR1 distance (m) × 0.0084 + 36.4 [37]. The ICC was evaluated for reliability with test–retest and found to be 0.81.

### Statistical analyses

Statistical analyses were performed using GraphPad Prism 8.0.1. The significance level was set at P < 0.05, a priori, and data were presented as mean and SD. Shapiro–Wilk was applied to check the normality the data. Pearson correlation analysis was performed between the maturity statues, physical fitness tests, and IGF1 with MP. While Spearman correlations were used for GH and soccer training. The following thresholds were used to define the strength of the correlation coefficients [49]: < 0.1 = trivial; 0.1–0.3 = small; 0.3–0.5 = moderate; 0.5–0.7 = large; 0.7–0.9 = very large; and > 0.9 = nearly perfect. Following that, using the variables listed above, linear and multiple linear regression were used to see how well MP could be predicted. We analyzed the ICCs for all measures' within-session reliability, but we analyzed the IR1 test–retest reliability. The ICC value of 0.7 was appropriate [50].

## Results

Descriptive characteristics of players are presented in Table 2, where values are reported as mean ± SD. The total season consisted of 1383.37 ± 260.76 min of playing, over 33 matches.

**Table 2:** Descriptive characteristics of players

Variables	Mean ± SD
Height (cm)	176.50 ± 5.23
Weight (kg)	60.97 ± 8.29
Chronological age (yrs)	15.61 ± 0.27
Skeletal age (yrs)	16.65 ± 0.84
Maturity Offsets (yrs)	1.97 ± 0.43
Soccer training (mo)	79.92 ± 19.48
V̇O_2max_ (ml kg^−1^ min^−1^)	49.84 ± 3.48
Best sprints (s)	6.44 ± 0.24
Worst sprints (s)	7.00 ± 0.31
Fatigue index (s)	0.56 ± 0.29
CMJ (cm)	40.87 ± 4.24
GH (ng/dl)	1.77 ± 2.21
IGF1 (ng/dl)	358.33 ± 105.57
Minutes of playing (min)	1383.37 ± 260.76

V̇O_2max_: maximal oxygen consumption; CMJ = countermovement jump; GH = growth hormone; IGF1 = insulin-like growth factor

**Fig. 1:**
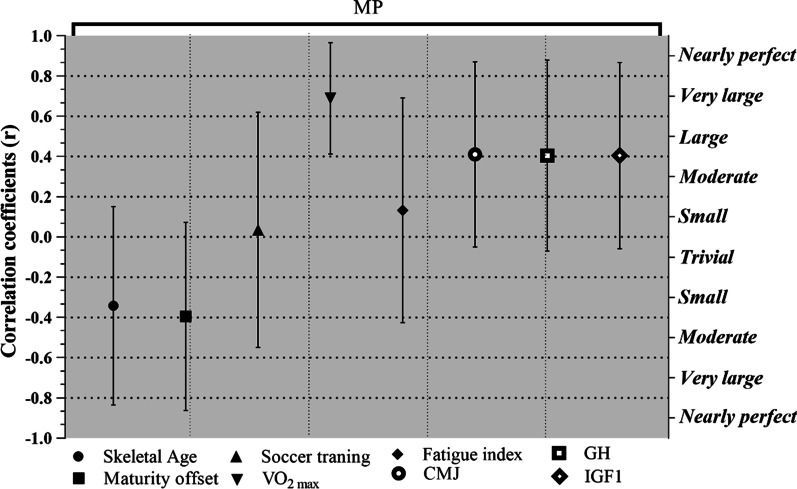
Correlation coefficients (95% CI) the minutes of playing soccer player in the compositions with the skeletal age (yrs); maturity offset (yrs); Soccer training (mo); VO_2max_ (ml g^−1^ min^−1^); Fatigue index (s); CMJ indicates the best of countermovement jumps performance (cm); GH, and IGF1 levels (ng/dl)

Figure 1 depicts the correlations between MP and maturation status, fitness status, and hormonal levels. Accordingly, there were significant correlations between MP and Maturity offset (r = − 0.46; CI 95% [− 0.73 to − 0.06]; *p* = 0.02), CMJ (r = 0.47; CI 95% [0.8 to − 0.73]; *p* = 0.02), GH (r = 0.47; CI 95% [0.07 to 0.74]; *p* ≤ 0.02), IGF1 Level (r = 0.47; CI 95% [0.07 to 0.73]; *p* ≤ 0.02), and V̇O_2max_ (r = 0.75 large; CI 95% [0.49 to 0.88]; *p* ≤ 0.0001). In addition, there were significant correlations between V̇O_2max_ and maturity offset (r = − 0.52; CI 95% [− 0.76 to − 0.14]; *p* = 0.009), CMJ (r = 0.53; CI 95% [0.16 to 0.77]; *p* = 0.008), and skeletal age (r = − 0.41; CI 95% [− 0.70 to − 0.01]; *p* = 0.04) (Table 3).

**Table 3:** Pearson and spearman correlation analysis

Variable	β0	β1	β2	β3	β4	β5	β6	β7	β8
MP (β0)	1.00								
Skeletal Age (β1)	− 0.40	1.00							
Maturity offset (β2)	− **0.46**	0.36	1.00						
Soccer training (β3)	0.43	0.03	− 0.11	1.00					
V̈O_2max_ (β4)	**0.75**	− **0.41**	− **0.52**	0.21	1.00				
Fatigue index (β5)	0.16	− 0.18	− 0.38	− 0.12	− 0.02	1.00			
CMJ (β6)	**0.47**	− 0.08	− 0.16	− 0.04	**0.53**	0.06	1.00		
GH Level (β7)	**0.47**	− 0.32	0.03	− 0.05	0.33	− 0.15	0.30	1.00	
IGF1 Level (β8)	**0.47**	− 0.33	0.06	− 0.12	0.26	0.25	0.21	0.16	1.00

Significant differences (p ≤ 0.05) are highlighted in bold

MP minutes of playing, CMJ countermovement jumps. MP = minutes of playing; V̇O_2max_ = maximal oxygen consumption; CMJ = countermovement jump; GH = growth hormone; IGF1 = insulin-like growth factor

**Fig. 2:**
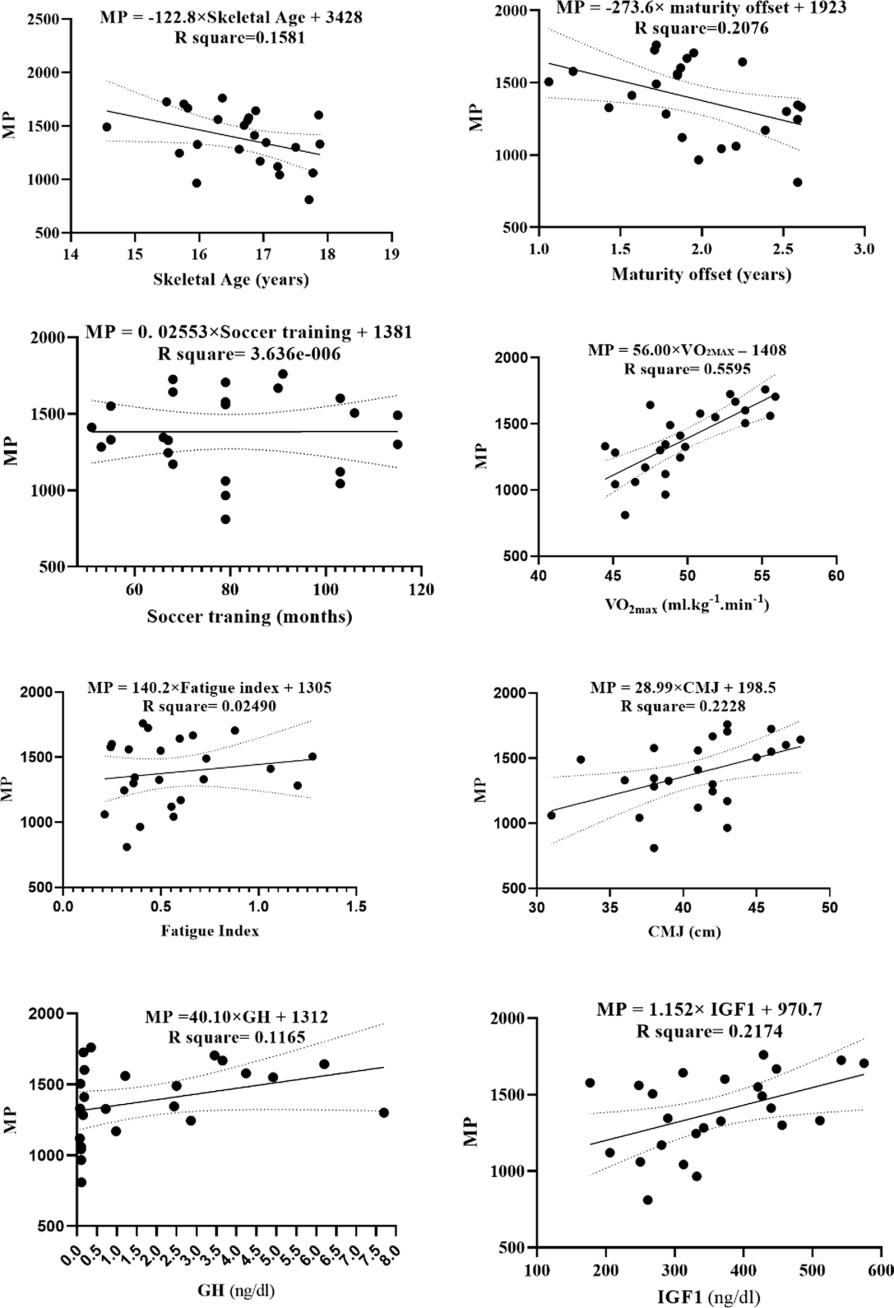
Regression analysis to explain MP (the minutes playing in each competitive level). MP = minutes of playing; VO_2max_ = maximal oxygen consumption; CMJ = countermovement jump; GH = growth hormone; IGF1 = insulin-like growth factor

**Fig. 3:**
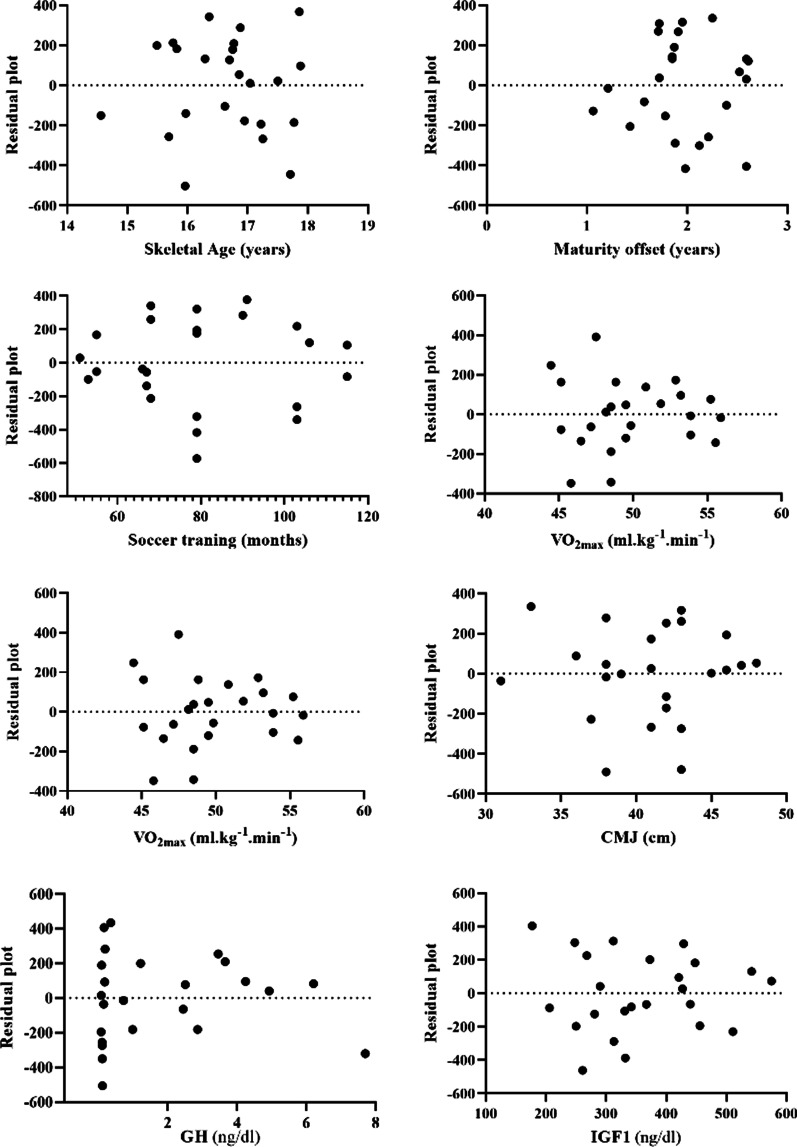
Residual plot; the difference between the actual value of the dependent variable and the value predicted by the residual provided**.** MP = minutes of playing; VO_2max_ = maximal oxygen consumption; CMJ = countermovement jump; GH = growth hormone; IGF1 = insulin-like growth factor

Linear regression models are depicted in Fig. 2, and demonstrate that MP is significantly predicted by the level of maturity offset (F (1, 22) = 5.77, b = − 273.6, *p* = 0.03), with an R^2^ = 0.21; V̇O_2max_ (F (1, 22) = 27.95, b = 56.00, *p* ≤ 0.0001), with an R^2^ = 0.56; CMJ (F (1, 22) = 6.31, b = 28.99; *p* = 0.02) with an R^2^ = 0.22; GH (F (1, 22) = 2.90, b = 40.10; *p* = 0.015) with an R^2^ = 0.1165, and IGF1 levels (F (1, 22) = 6.11, b = 1.15; *p* = 0.02, R^2^ = 0.22, respectively. MP was increased by 56.00 min for each ml.kg^−1^.min^−1^ of VO_2max_; 28.99 min for each cm of CMJ; 40.10 min for each ng/dl of GH, and increased 1.15 min for each ng/dl of IGF1. Conversely, MP decreased − 273.6 min for each year of maturity offset. For clarity, the residual plots are shown in Fig. 3.

Multiple linear regression analysis highlighted that maturation status, fitness status, and hormonal levels could significantly predict MP (F (8, 15) = 6.05, *p* = 0.001), with an R^2^ of 0.76, and derived as MP (Y) = -Beta0 + Beta1 (Skeletal Age)—Beta2 (Maturity offset)—Beta3 (Soccer training) + Beta4 (V̇O_2max_) + Beta5 (Fatigue index)—Beta6 (CMJ) + Beta7 (GH) + Beta8 (IGF1). It was found that MP increased 37.91 min for each ng/dl of GH, and increased 42.83 min for each ml.kg^−1^.min^−1^ of V̇O_2max_.

Both GH and V̇O_2max_ were significant predictors of MP (Table 4). Accordingly, another predictive equation was made for these two variables, which significantly predicted MP (F (2, 21) = 17.92, *p* < 0.0001), with an R^2^ of 0.63. Our findings highlighted that for each ml.kg^−1^.min^−1^ of V̇O_2 max_ or each ng/dl of GH, MP increased by 53.95 and 31.46 min, respectively.

**Table 4:** Multiple linear regression analysis: minutes of playing and all variables

Variable	Beta	Estimate	|t|	P value	95% CI for estimated
MP (min)	β0	− 904.3	0.77	0.455	− 3415 to 1607
Skeletal Age (years)	β1	20.17	0.43	0.676	− 80.63 to 121.0
Maturity offset (years)	β2	− 160.6	1.40	0.181	− 404.7 to 83.59
Soccer training (months)	β3	− 1.9	1.09	0.292	− 5.806 to 1.870
V̇O_2 max_ (ml kg^−1^ min^−1^)	β4	42.8	2.72	**0.016**	9.324 to 76.35
Fatigue index (seconds)	β5	40.3	0.28	0.780	− 262.2 to 342.8
CMJ (cm)	β6	− 1.2	0.12	0.907	− 22.55 to 20.17
GH (ng/dl)	β7	37.9	2.32	**0.035**	3.034 to 72.78
IGF1 (ng/dl)	β8	0.7	1.81	0.091	− 0.1250 to 1.519
				R^2^ = 0.76	

Significant differences (p ≤ 0.05) are highlighted in bold. MP (minutes of playing); CMJ (countermovement jumps)

MP = minutes of playing; V̇O_2max_ = maximal oxygen consumption; CMJ = countermovement jump; GH = growth hormone; IGF1 = insulin-like growth factor; CI = confidence interval

## Discussion

In the present study, we sought to investigate the relationship between playing time and maturation, physical performance, and hormonal levels. The relationship between maturity status, hormonal levels and physical performance has been investigated previously, revealing equivocal findings [21, 51]. We have hypothesized that the magnitude of maturity status and physical fitness level of performance would dictate the playing time of U16 team. Indeed, in our study, we found very large correlations between V̇O_2max_. and MP, and moderate correlations between GH, IGF1, maturity offset, CMJ, and MP. Moreover, large correlations were found between V̇O_2max_ and maturity offset, and CMJ, respectively.

Children’s peak general fitness development is often coincident with PHV, which occurs at approximately 14 years of age [51]. Indeed, in a study conducted using children aged 11 to 15 years old, it was revealed that peak development of aerobic, speed, and agility performance occurred at the same time as the peak height spurt between 13 and 14 years of age [52]. However, in the same study, the authors revealed that peak development of strength occurred at approximately 15–16 years of age. Concordantly, it was somewhat expected that, in our sample, the players had greater physical qualities due to maturity, as they presented with a mean chronological age of 15 years, and a mean biological age of 16 years. Further, this may suggest that players with greater levels of V̇O_2max_ are better at power-related performances, thus, representing a determining factor regarding selection for competition and greater minutes of play.

A linear regression for each of the independent variables was conducted to investigate their influence on MP. Indeed, V̇O_2max_, CMJ, maturity offset, IGF1, and GH could account for 56%, 22%, 21%, 21%, and 11% of the variation in MP, respectively. Despite V̇O_2max_ in the present study seemed to also influence the MP, it must be noted that V̇O_2max_ measure was estimated from the YYIR level 1, which consists in a field test that measures aerobic performance [48, 53].

The GH-IGF1 axis is well known to have a great impact on linear and normal growth development [15]. Indeed, during late puberty, the levels of GH and IGF1 increase significantly, and peak at approximately between 15–16 years of age [54]. Given that IGF1 concentration levels are reported to be higher than GH levels at this stage of puberty (our sample is at ~ 16 of biological age), this may plausibly account for why IGF1 exerted a greater influence on MP than GH. Moreover, because operating hormone activity is associated with maturity status during puberty, it is conceivable that these anabolic pathways may lead to greater physical performance [18, 54]. Furthermore, programs of soccer training imposed on U16 appear to increase GH and IGF1 activity and concentrations, which consequently, yield concomitant increases in strength levels and aerobic performance [18]. Given that post-PHV, the GH and IGF1 levels return to normal concentrations therein reducing the anabolic environment, it is conceivable that strength and power characteristics might influence MP less than aerobic capacity [21, 32, 54]. However, from the findings of our study, it appears that hormonal activity, fitness levels, and maturity offset may influence the selection for competition and MP.

In the present study, only a moderate relationship between skeletal age and predicted PHV was observed. In fact, a study conducted on 143 youth soccer players, with the aim to validate the estimation of PHV relative to skeletal age, found only a moderate correlation between skeletal age (assessed via the Fels method) and the PHV estimation method [55]. Similarly, a recent study conducted on 71 youth athletes found that the associations between skeletal age method and the predicted age at PHV method were poor [56]. The reasons behind the lack of stronger relationships between these two methods can be attributed to the fact that skeletal age measures the maturity status of skeleton at the assessment moment, while maturity offset is a prediction based on timing [6]. Also, the PHV estimation via maturity offset calculation is dependent on chronological age [10]. Beside it is important to keep in mind that different timings of growth in a group of athletes can impact the body size and may cause an influence on the associations between the skeletal age and predicted PHV methods [56].

Compared to linear regression models, multiple linear regressions are able to yield more insight into what is influencing the dependent variable (minutes of play), by accounting for multiple factors (maturity, hormonal, and physical dimensions) [57]. Accordingly, the multiple linear regression analysis revealed that the interaction of the independent variables explains 76% of MP. Further, when analyzing the independent variables alone, we found that GH and V̇O_2max_ explain 63% of MP, representing the most significant predictors of MP in this study. In a previous study, conducted on 29 elite young soccer players, that sought to predict the factors influencing MP through a multiple linear regression, it was revealed that 16.7% of the variance in the MP in a match was explained by jump performance, which is discordant with our results. Indeed, as previously highlighted, the evidence ascertained in the present study suggests that GH levels and aerobic capacity, together, may have greater importance in the selection for competition and MP than jump performance. However, although these two explanatory variables appear to more strongly influence MP, caution should be taken when interpreting these findings, and should be considered in the context of all the included independent variables.

Although the present study represents a novel contribution to the literature, our study has some limitations that should be considered. The sample size was one of the main limitations, because only one team was analyzed. However, as elite youth soccer teams have time and training restrictions as found in professional adult teams, gathering volunteers of other teams is impractical [1]. Moreover, technical and tactical factors were not considered, as well as the possible influence of positional related tasks on selection and MP. For those reasons, future studies should consider using larger sample sizes, including technical/tactical and positional dependencies, to potentially yield more robust information about selection and MP.

To the authors knowledge, this was the first study that analyzed the influence of maturity status, hormonal activity, and physical variables, collectively, on the selection for competition and MP. The integration of hormonal activity, fitness levels, and maturation status revealed a meaningful influence on MP, with V̇O_2max_ and GH being the most preponderant factors for selection and MP. Additionally, aerobic capacity, jump performance, and maturity offset demonstrated significant correlations with MP. Thus, the integration of these explanatory variables, collectively, may yield more detailed information about what influences the selection for competition and the MP in elite youth soccer.

As practical applications, both coaches and practitioners would benefit more to use aerobic capacity, jump performance, and hormonal status of each player to design more individualized training programs with the intent to develop the players with lower values. This can potentially lead coaches to have more players available to be selected and improve within-team competition, and avoiding the selection of always the same players. That is, as more advanced in maturity and in physical performance players seem to be the ones that have more MP, developing the players with less advanced maturity and physical performance will increase competitive fairness within the same team.

## Conclusions

In conclusion, large correlation coefficients were evident between MP and aerobic performance, and between V̇O_2max_ and maturity offset and CMJ, respectively. We found that the interaction of maturity, hormonal, and physical dimensions accounted for 76% of MP, whilst GH and V̇O_2max_ were the strongest determining factors influencing MP. Taken collectively, at later stages of puberty, aerobic performance appears to have an important role MP. However, the integration of maturity, hormonal, and physical dimensions may yield more robust information.

## Data Availability

The datasets generated and analysed during the current study are not publicly available due to ethical restrictions, however, they are available from the corresponding author on reasonable request.

## Abbreviations

GH: Growth hormone
IGF-1: Insulin-like growth factor-1
SA-CA: Skeletal age and chronological age
PHV: Peak height velocity
CMJ: Countermovement jump
7RST: Seven repeated sprint test
SD: Standard deviation
MP: Minutes of playing
V̇O_2max_: Maximal oxygen consumption
